# MicroRNAs, Hypoxia and the Stem-Like State as Contributors to Cancer Aggressiveness

**DOI:** 10.3389/fgene.2019.00125

**Published:** 2019-02-20

**Authors:** Lucy Wanjiku Macharia, Caroline Muriithi Wanjiru, Marianne Wanjiru Mureithi, Claudia Maria Pereira, Valéria Pereira Ferrer, Vivaldo Moura-Neto

**Affiliations:** ^1^Instituto Estadual do Cérebro Paulo Niemeyer – Secretaria de Estado de Saúde, Rio de Janeiro, Brazil; ^2^Programa de Pós-Graduação em Anatomia Patológica, Faculdade de Medicina da Universidade Federal do Rio de Janeiro, Rio de Janeiro, Brazil; ^3^Instituto de Ciências Biomédicas da Universidade Federal do Rio de Janeiro, Rio de Janeiro, Brazil; ^4^KAVI Institute of Clinical Research, Faculty of Medicine, University of Nairobi, Nairobi, Kenya; ^5^Laboratório de Genética, Universidade do Grande Rio, Duque de Caxias, Brazil

**Keywords:** microRNAs, hypoxia, stem-like state, cancer, cancer aggressiveness, microenvironment

## Abstract

MicroRNAs (miRNAs) are small non-coding RNA molecules that play key regulatory roles in cancer acting as both oncogenes and tumor suppressors. Due to their potential roles in improving cancer prognostic, predictive, diagnostic and therapeutic approaches, they have become an area of intense research focus in recent years. Several studies have demonstrated an altered expression of several miRNAs under hypoxic condition and even shown that the hypoxic microenvironment drives the selection of a more aggressive cancer cell population through cellular adaptations referred as the cancer stem-like cell. These minor fractions of cells are characterized by their self-renewal abilities and their ability to maintain the tumor mass, suggesting their crucial roles in cancer development. This review aims to highlight the interconnected role between miRNAs, hypoxia and the stem-like state in contributing to the cancer aggressiveness as opposed to their independent contributions, and it is based in four aggressive tumors, namely glioblastoma, cervical, prostate, and breast cancers.

## Introduction

About 70–80% of the human genome is transcribed into RNA and only about 2% of the human genome constitutes protein-coding genes meaning that the rest of the genome contains more non-coding genes mostly referred to as non-coding RNA (ncRNA). Additionally, ncRNAs include long non-coding RNAs (lncRNA), PIWI-interacting RNAs (piRNA), small interfering RNAs (siRNA) and microRNAs (miRNA) among others (Alexander et al., 2010; Sana et al., 2012; Qureshi and Mehler, 2012; Kartha and Subramanian, 2014). However, approximately 60% of all protein-coding genes are potentially regulated by miRNAs, which confers them a fundamental role in the modulation of numerous cell processes and disorders, such as cancer (Bartel, 2009; Ma et al., 2012). Several miRNAs play important roles during hypoxia, an essential feature of the neoplastic microenvironment (Harris, 2002; Vaupel et al., 2004) and the molecular mechanisms responsible for the hypoxic survival of neoplastic cells are not fully characterized. A better understanding of this process may lead to novel strategies for pharmacological intervention. Moreover, hypoxia has been associated with maintenance of an undifferentiated cell state (Parmar et al., 2007; Clarke and van der Kooy, 2009; Silván et al., 2009) and several studies have shown that restricted oxygen conditions expand the fraction of cells positive for a cancer stem cell marker (Tavaluc et al., 2007; Das et al., 2008; McCord et al., 2009). Hypoxia and the stem-like state influence the expression profile of a set of miRNAs and the same miRNAs play prosurvival roles in this microenvironment and in the maintenance of the tumor cell in its precursor state. Various studies show that miRNAs, the stem-like state and the tumor microenvironment all play important roles in the tumor invasiveness, but their interconnected roles will be further explored in this article.

## MicroRNAs

**FIGURE 1:**
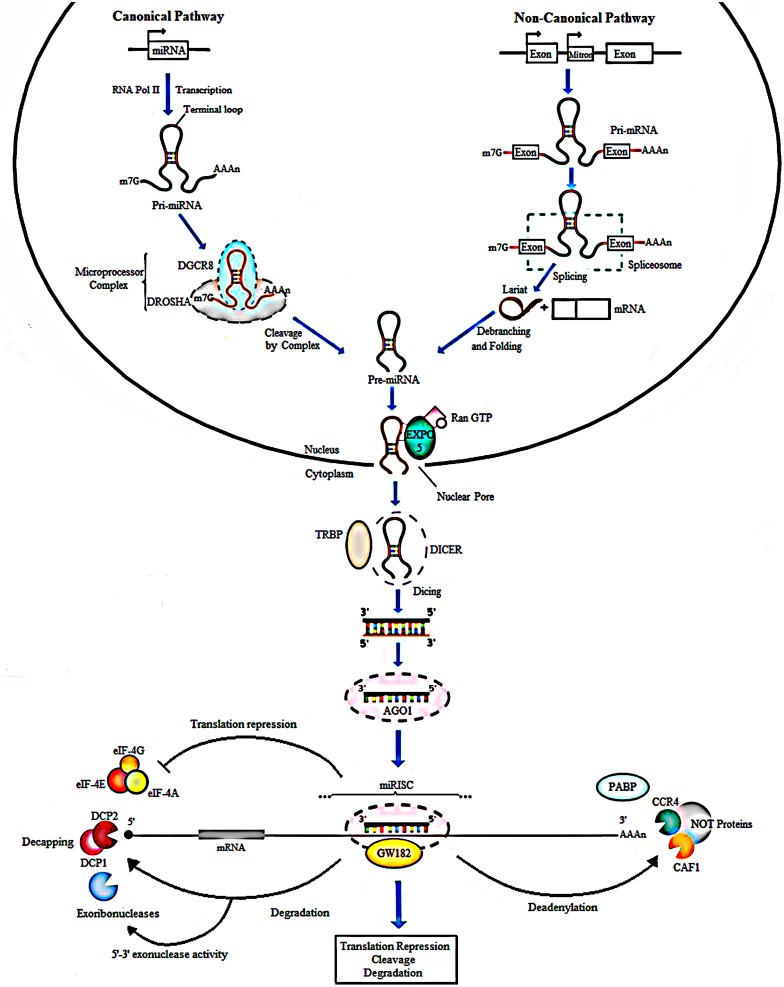
Biogenesis of miRNAs. In the canonical pathway, miRNAs are transcribed from their loci by RNA polymerase II into a long primary transcript of about 80 nucleotides called the pri-miRNA. Cleavage follows and is done by Drosha, a type III RNase along with the DGCR8 protein to produce pre-miRNA. In the non-canonical pathway, Mirtrons are spliced by the spliceosome to form looped intermediates referred to as lariat which then refold into pre-miRNAs. Next, the exportin 5, a RAN-GTP dependent transporter, mediates the movement of pre-miRNAs from the nucleus into the cytoplasm. Further processing by Dicer and TARBP2 protein generates mature miRNAs, producing double-stranded structure of miRNA of about 21-22 nt in length. The duplex is loaded into an AGO protein. The passenger strand (miRNA^∗^) is degraded, whereas the guide strand is incorporated by the Ago into the miRNA-induced silencing complex (miRISC). In animals, imperfect complementarity occurs when the miRNA seed region, nucleotides 2-8, BPs perfectly with the complementary seed match site in the 3′ UTR of the target mRNA resulting in translational repression or degradation. GW182 a core component of miRISC, mediates deadenylation of mRNAs by interacting with AGO and PABP consequently leading to recruitment of deadenylases like CCR4 and CAF1. Translation repression can result from inhibited binding of PABP to the poly (A) tail of the mRNA, responsible for attracting the elFs to mRNA to initiate translation. The formation of the CCR4-CAF1-NOT complex, a poly A tail-truncating enzyme, mediated by binding of miRISC to mRNA results in truncation of the downstream poly A tail, reduced binding of translation initiation factors and translation repression. The shortening or complete removal of the poly (A) tail induces the removal of the 5′ cap of the mRNA. Decapping is also mediated by DCP1 and DCP2. Consequently, the uncapped mRNA is rapidly degraded by 5′-3′ exoribonucleases.

MicroRNAs are small non-coding RNAs composed of approximately 21–22 nucleotides (nts) and were first discovered in *Caenorhabditis elegans* (Lee et al., 1993). Their biogenesis generally starts from transcription of intergenic, intronic or polycistronic loci by RNA polymerase II (Lee et al., 2004; Borchert et al., 2006) to a 80-nts, capped, polyadenylated pri-miRNA transcripts bearing a stem-loop structure (Figure 1). In the canonical pathway, nuclear RNase III- Drosha, in complex with dsRBPs, for example DGCR8 and transactivation-responsive (TAR) RNA-binding protein (TRBP) in mammals, cleaves the pri-miRNA to give rise to a 70-nts long RNA molecule with a 2-nt overhang at the 3′ end called pre-miRNA. Besides the canonical miRNA biogenesis pathway, many Drosha–DGCR8- independent pathways produce pre-miRNAs. The most common alternative pathway involves short intronic hairpins termed mirtrons, that are spliced by spliceosome to form looped intermediates, lariat, which then refold into the pre-miRNA hairpins (Ruby et al., 2007; Yang and Lai, 2011; Ladewig et al., 2012; Palanichamy and Rao, 2014). After, the pre-miRNA is exported to the cytoplasm by Exp5, a Ran-GTP dependent nucleo/cytoplasmic cargo transporter (Yi, 2003). Its 3′ overhang is then recognized by Dicer another RNase III enzymes, in complex with transactivation- responsive RNA-binding protein 2 (TARBP2) and binds it to its PAZ domain to cleave the terminal loop, resulting in a 21- to 22-nt double-stranded RNA that contain a 2-nt overhang on both ends termed as mature miRNA (Bernstein et al., 2001; Grishok et al., 2001; Lee et al., 2003; Yi et al., 2003; Bohnsack et al., 2004; Denli et al., 2004). In the cytoplasm, miRNA duplexes are incorporated into an Argonaute (Ago) protein containing miRISC followed by unwinding of the duplex (miRNA/miRNA^∗^ duplex) and retention of the mature miRNA strand (guide strand) in miRISC, while the complementary strand (passenger or miRNA^∗^) is released and degraded (Carthew and Sontheimer, 2009; Krol et al., 2010) (Figure 1).

Different levels of complementarity between the miRNA and mRNA can lead to various effects on gene expression. That is, if the complementarity is perfect, miRNA function as short interfering RNA (siRNA) and the target mRNA is sequence-specifically cleaved by the miRISC complex. However, this occurrence is rare in animals and imperfect complementarity happens where the miRNA seed region, nucleotides 2–8 in the mature miRNA, base pairs (BPs) perfectly with the complementary seed match site in the 3′untranslated region (3′ UTR) of the target mRNA (Bartel, 2009) consequently leading to translational repression or mRNA degradation (Krol et al., 2010; Huntzinger and Izaurralde, 2011). Further, glycine-tryptophan protein of 182 kDa (GW182), a core component of miRISC, mediates deadenylation of mRNAs by interacting with AGO and poly (A) binding protein (PABP) consequently leading to recruitment of deadenylases like CCR4 and CAF1 (Eulalio et al., 2009; Fabian et al., 2010). The formation of the CCR4-CAF1-NOT complex, a poly A tail-truncating enzyme, mediated by binding of miRISC to mRNA, results in truncation of the downstream poly A tail, reduced binding of translation initiation factors and translation repression (Wu et al., 2006; Standart and Jackson, 2007; Torres et al., 2011). The shortening or complete removal of the poly (A) tail induces the removal of the 5′ cap of the mRNA. Consequently, the uncapped mRNA is rapidly degraded by 5′-3′ exoribonucleases (Treiber et al., 2012) (Figure 1).

### miRNAs and Cancer Aggressiveness

In this article, tumor aggressiveness is a term used to refer to a highly invasive, incurable, end stage cancer associated with therapy resistance and a poor patient prognosis (Lima et al., 2012; Arpino et al., 2015; Small et al., 2017).

**FIGURE 2:**
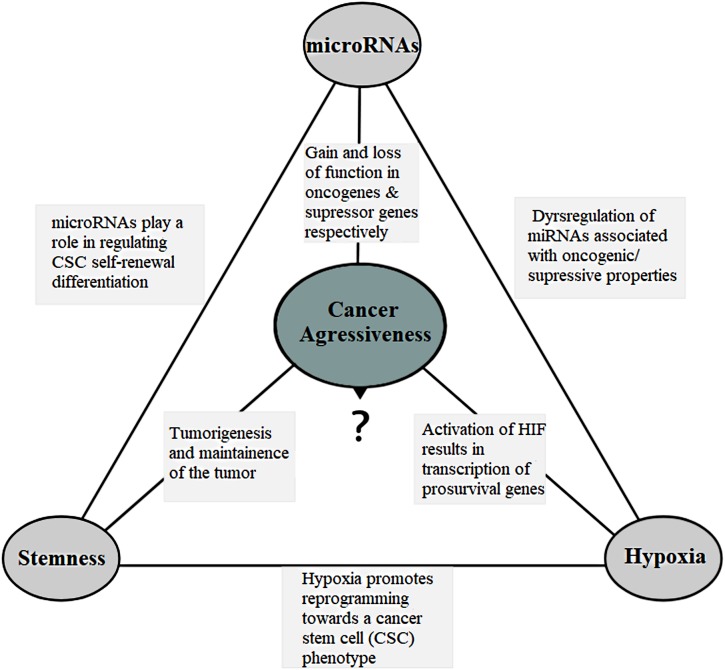
The connection triangle. The miRNAs, hypoxia and the stem-like state all play a connected role that eventually culminates in the tumor aggressiveness. miRNAs and aggressiveness: miRNAs are often found dysregulated and have gain-of-function mutations in the miRNAs associated with the oncogenic property and loss-of-function mutations in the miRNAs with the tumor suppressor properties. miRNAs and stemness: miRNAs play a role in the regulation of cell self-renewal, differentiation and regulation of transcription factors including Nanog, SOX2 and OCT4 among others associated with the stem-like state. They also help the stem-like cells to override the G1/S checkpoint to sustain continual division. miRNAs and hypoxia: miRNAs assist forming hypoxic microenvironment and regulate the HIF switch during hypoxia. Switch of HIF-1 to HIF-2 and HIF-3 is required in order to adapt the cells to prolonged or chronic hypoxia that may otherwise lead to apoptosis. Hypoxia and aggressiveness: In intratumoral hypoxia, a phenomenon of the aggressive cancers, hypoxia inducible factor 1α (HIF-1α) is stabilized, and in turn, HIF-1 results in transcription of pro-survival target genes involved with tumor angiogenesis, invasion, cell survival, EMT, and metabolism among others. Hypoxic pseudopalisading zones are protected from chemoradiation because of vascular stasis and depletion of molecular oxygen cells contributing to aggressiveness. Hypoxia and miRNAs: under hypoxia, a group of miRNAs, hypoxia regulated miRNAs-HRMs, are deregulated modulating processes involved in tumor survival. Hypoxia and stemness: Hypoxia promotes reprogramming towards a cancer stem-like cell (CSC) phenotype and expansion of CSC populations, a population thought to be responsible for the maintenance and recurrence of the tumor. It is in the hypoxic regions also that the CSCs go into quiescence to escape from targeted therapy. Stemness and aggressiveness: It is believed that stem-like cells have the capacity to sustain tumorigenesis by maintaining the tumor growth. They also contribute to therapy resistance, invasion and metastasis. Stemness and miRNAs: Evidences have shown that stem-like cells fine tune the miRNA expression. This includes “inhibitory-like” role of the miRNAs associated with tumor differentiation to restore “stemness” a probable source for tumor recurrence.

miRNAs are dysregulated in almost all human cancers (Di Leva et al., 2014; Palanichamy and Rao, 2014) and can function as either oncogenes (oncomirs) or tumor suppressors (anti-oncomirs), depending on their target transcripts (Figure 2). In the canonical model, oncomirs are upregulated and anti-oncomirs are downregulated, which has been attributed to amplification, deletion and/or mutation of miRNA loci, dysregulation of transcription factors and epigenetic silencing (Calin et al., 2004; Zhang et al., 2006). Globally, miRNA expression alterations lead to the regulation of several oncogenic or tumor-suppressor protein levels, which in turn alter cell behavior favoring tumor aggressiveness (Zhang et al., 2007; Iorio and Croce, 2012).

Various studies on individual miRNA function or expression in glioblastoma (GB), cervical (CC), prostate (PCa) and breast cancers (BC), have established that miRNAs play a crucial role in different aspects of tumorigenesis. Specifically, miRNAs mediated mechanisms regulate a wide range of functions including cell viability, cell proliferation (Cui et al., 2010), cell migration and invasion (Zhang et al., 2010; Bao et al., 2018), apoptosis (Shi et al., 2011), tumor growth (Zhu et al., 2018), cell cycle (Peng et al., 2018), chemo- and radio resistance (Ke et al., 2013; Wang W. et al., 2016), angiogenesis (Yue et al., 2012), tumor metabolism (Liu Z. et al., 2017), epithelial-mesenchymal transformation (Peng et al., 2011), and maintenance of the stem-like state (Shi et al., 2010, 2014; Yang et al., 2015; Wang et al., 2017) among others that culminates to cancer aggressiveness. There are clinical studies focused on assessing miRNA expression patterns to determine its value as a therapeutic, prognostic and diagnostic marker using patient samples. Example of such clinical trials is a study correlating miR-10b with gliomas (ClinicalTrials.gov identifier: NCT01849952). Elsewhere, a study that aimed to evaluate the expression levels of miR-10b in breast cancer patients, showed miR-10b expression to be correlated with disease stage, living status and the tumor size (Zhang et al., 2018). Various other studies linking miRNA and patient prognosis or the clinical response in various diseases are summarized by Nana-Sinkam and Croce (2013) and Monroig-Bosque et al. (2015).

### miRNAs, Epigenetic Mechanisms and Cancer Aggressiveness

In as much as miRNAs are dysregulated in a variety of cancers, the mechanisms behind their dysregulation are unclear. Research advances have lead to the knowledge that epigenetic alterations, including aberrant DNA methylation and histone modifications, are major causes of miRNA dysregulation in cancer (Suzuki et al., 2013). DNA methylation is mediated by the activation of the enzyme DNA methyltransferase (DNMT) and specifically (DNMT1, DNMT3A, and DNMT3B) have the catalytic methyltransferase activity of which DNMT1 is the most abundant DNMT in mammalian cells (Veeck and Esteller, 2010). These enzymes act at the cytosine that is in general adjacent to the guanine, converting that to 5-methylcytosine (5-mC). Oncogenic miRNAs are now known to be upregulated via DNA hypomethylation while tumor suppressors are silenced via DNA hypermethylation of CpG island-associated gene promoters, thereby facilitating the initiation and progression of cancer (Veeck and Esteller, 2010; Suzuki et al., 2013; Morales et al., 2017). Some works have shown the hotspots for miRNA methylation in human chromosomes 1, 2, 9, 11, 16, 17, and 19 (Kunej et al., 2011; Morales et al., 2017). Similarly, a higher than expected proportion of miRNA genes has been found in the CpG islands susceptible to methylation (Gel et al., 2015). Recent evidence also show that miRNA expression can also be modulated by methylation of other regulatory regions, such as enhancers (Morales et al., 2017). Although CpG islands, are located in the promoters of 60% of the protein coding genes in human genome, the frequency of human miRNA gene methylation is higher than the protein-encoding genes (Weber et al., 2007).

The common types of histone modifications are lysine (K) acetylation and lysine methylation. While lysine acetylation mediated by histone acetyltransferases (HAT) usually uncoils the chromatin structure and enhance transcription activation, deacetylation mediated by histone deacetylases (HDAC) removes the acetyl groups provoking chromatin condensation and gene inactivation. Lysine methylation might generate different effects on gene expression depending on the position and degree of methylation. Methylation at H3K4, K36 and K79 has been associated with gene activation, whereas methylation at H3K9, H3K27 and H4K20 has been correlated with transcriptional repression (Zhou et al., 2011; Tessarz and Kouzarides, 2014; Romani et al., 2018).

miR-101 is down regulated in GB and it has been shown to reverse CPEB1 (hypomethylation gene) promoter methylation status by regulating the methylation-related histones H3K27me3, H3K4me2, H4K20me3, and H3K9me3 in GB (Li and Wu, 2017). Similarly, methylation-associated silencing of miR-34c has been shown to promote self-renewal and epithelial–mesenchymal transition in breast tumor- initiating cells (Yu et al., 2012). Moreover, DNA methylation and/or EZH2-mediated histone methylation were recently confirmed to contribute to miR-31 loss in TNBC (Augoff et al., 2012). Elsewhere, miR-155 has been shown to be epigenetically repressed by wild-type gene BRCA1 through its interaction with HDAC2 resulting in deacetylation of H2A and H3 on the miR-155 promoter in normal breast tissues (Chang et al., 2011). JARID1B demethylase has been shown to contribute to breast cancer cell proliferation through the epigenetic repression (removing the active mark H3K4me3) of let-7e a tumor suppressor miRNA. Additionally, JARID1B can also repress miR-1246, miR-1826 and miR-361-5p (Mitra et al., 2011). Similarly, a study using PCa cells identified miR-615 as an epigenetically activated miRNA through the loss of DNA methylation and gain of H3K9 acetylation (Hulf et al., 2011). As it is beyond the scope of this review to exhaust all the epigenetic mechanisms that regulate the progression of cancers and miRNA dysregulation, detailed explanations have been summarized by Rennie and Nelson (1999), Liu et al. (2013), Suzuki et al. (2013), Dai et al. (2014), Fang et al. (2014), Karsli-Ceppioglu et al. (2014), Morales et al. (2017), and Ruggero et al. (2018).

### Protein-Coding Genes Modifications and Cancer Aggressiveness

The accumulation of genetic mutations has been considered the major cause of neoplasia (Hanahan and Weinberg, 2011) whose path is relatively straightforward: mutation of tumor suppressors and/or oncogenes causing either the loss or gain of functions and abnormal expression (You and Jones, 2012).

Recently, gene expression profiles have been translated into clinical applications either as prognostic or predictive signatures in cancer. A study using pathway and machine learning based methods found a 35-gene expression signature that can discriminate between rapidly- and slowly progressing GB and its prognostic value (Fatai and Gamieldien, 2018). Similarly, a 21-gene assay, Oncotype DX, has been used to predict likelihood of recurrence in women with ER positive breast cancer (Paik et al., 2004; Goldstein et al., 2008). Gene expression analysis using MammaPrint Symphony, a 70-gene panel, enables a fairly dynamic assessment of neoplastic process, allowing categorizing breast cancer patients according to the high or low risk for relapse (van ’t Veer et al., 2002; Bueno-de-Mesquita et al., 2009).

Several genetic lesions including TP53, EGFR and PTEN mutations have long been identified in GB (Brennan et al., 2013). Sequencing of over 20,000 genes discovered an extended number of altered protein coding sequences including IDH1 (isocitrate dehydrognase) that were altered in more than 10% of GB (Parsons et al., 2008). Additionally, mutations in IDH1 are a hallmark molecular feature of secondary GB as compared to primary GB. Its occurrence is found in approximately 70% of secondary GB and 5–20% of primary GB (Ichimura et al., 2009; Yan et al., 2009). The O(6)-methylguanine-DNA methyltransferase (MGMT), a DNA repair enzyme, whose frequency of MGMT promoter methylation has been shown to range from 30% to 60% in GB (Weller et al., 2010), is an independent favorable prognostic factor. Patients with methylated MGMT promoter tumors have a survival benefit when treated with Temozolomide (TMZ) (Hegi et al., 2005). TERT promoter mutations have been found in GBs where patients with TERT promoter mutations alone (i.e., no IDH mutation) were found to have the poorest overall survival (Killela et al., 2013).

Persistent infection with high-risk human papilloma virus (HPV) entails an increased risk for development of CC (Walboomers et al., 1999). Functional loss of the tumor suppressor p53 by alterations in its TP53 gene is a frequent event in CC (Tommasino et al., 2003). Association of the viral oncoprotein E6 with the tumor suppressor p53 leads to degradation of p53 with subsequent inhibition of the transcriptional regulatory activities of the p53 protein (Thomas et al., 1999). Similarly, association of viral oncoprotein E7 with pRb, retinoblastoma tumor suppressor, also promotes the degradation of pRb (Jones et al., 1997) and disrupts the capacity of pRb to bind and inactivate functional cellular E2F transcription factors (Chellappan et al., 1992). A study using single-nucleotide polymorphism analysis, identified the IFNG, TMC6/8 (transmembrane channel-like 6 and 8) genes to be associated with CC progression (Wang et al., 2010).

A meta-analysis study has shown that mutations in the genes encoding glutathione S-transferase (GSTP1 and GSTM1) and G158A polymorphism in prostate-specific antigen gene have been associated with an increased risk for PCa (Gong et al., 2012). Similarly, BRCA1/2 mutations confer a more aggressive PCa phenotype with a higher probability of nodal involvement, distant metastasis and poor survival outcome in patients (Castro et al., 2013). Furthermore, high PSCA expression, expressed in the basal cells of normal prostate and in more than 80% of PCa, has been correlated with higher stage, metastasis, and poor outcome (Gu et al., 2000). Germline mutations in HOXB13 is associated with a significantly increased risk of hereditary PCa (Ewing et al., 2012). Equally important, deletion of the tumor suppressor gene encoding the PTEN has been described in more than two-thirds of patients with advanced/aggressive PCa. Genomic PTEN loss is associated with tumor progression and poor prognosis (Yoshimoto et al., 2007) and with increased risk of recurrence after prostatectomy for clinically localized PCa (Chaux et al., 2012).

A proportion of all breast cancers can be explained by the inheritance of germline mutations in one of the two major breast cancer susceptibility genes BRCA1 and BRCA2 related to DNA repair mechanisms (Claus et al., 1996; Martin and Weber, 2000). Similarly mutations in genes encoding other proteins that are involved in DNA repair expose patients to an elevated risk for breast cancer. In this sense, rare germline mutations in RAD51C a gene involved in the recombinational repair of double-stranded DNA breaks (Meindl et al., 2010) and in the ATM gene (Thompson et al., 2005), the cell cycle CHEK2 gene (Robson, 2009) have been shown to confer increased risks of breast cancer (Collins, 2011). Additionally, rare mutations in the PTEN (on chromosome 10) (Nelen et al., 1996) and STK11 (Boardman et al., 1998) genes associated with Cowden and Peutz–Jeghers syndromes, respectively, have been associated with an increased risk of breast cancer.

More information on known cancer susceptibility genes and regions have been summarized by Parsons et al. (2008), Li et al. (2016), and Bhargava et al. (2017) for GB (Bahrami et al., 2018; Tan et al., 2018) for CC (Collins, 2011; Michailidou et al., 2015) for BC, and (Martinez-Gonzalez et al., 2018; Benafif and Eeles, 2016) for PCa.

In conclusion, genetic alterations in the protein-coding genes play a huge role in the tumorigenesis of aggressive cancers where majority of these genes are potentially regulated also by epigenetically mechanisms, such as miRNAs. The pieces of evidence presented above are also in agreement that miRNAs play crucial roles in tumorigenesis and that epigenetic mechanisms play a major role in own miRNA dysregulation. Consequently, all these interwined mechanisms lead to cancer aggressiveness.

## Hypoxia Microenvironment

Hypoxia, low level of oxygen, is a fundamentally important phenomenon of solid tumors and specifically, intratumoral hypoxia is an important driving force for cancer progression that leads to patient mortality (Harris, 2002; Vaupel et al., 2004; Kulshreshtha et al., 2007). Hypoxic response is essentially mediated by hypoxia inducible factor-1alpha (HIF-1α) induced under conditions of low oxygen in a nuclear factor-κB-dependent manner (Jiang et al., 1997). At normal oxygen tension, HIF-1α is hydroxylated in the oxygen-dependent degradation domain (ODDD) by prolyl hydroxylases (PHD) and after recognized by the von Hippel–Lindau (VHL) protein and consequently ubiquitinated and selected for degradation by proteasome. This process is inhibited during hypoxia (Huang et al., 1996) where stabilized HIF-1α subunits heterodimerize with ß- subunits to form the active HIF-1 complex that consequently activates gene transcription by binding to the HRE; 5′-RCGTG-3′ in promoters and enhancers of target genes (Semenza, 1999).

HIF-1 regulates the transcription of hundreds of genes in response to hypoxia (Manalo et al., 2005; Elvidge et al., 2006) among which are glucose transporters, glycolytic enzymes, growth factors and genes involved in gluconeogenesis, high-energy phosphate and haem metabolism, erythropoiesis, iron transportation, vasomotor regulation and nitric oxide synthesis (Ratcliffe et al., 1998; Semenza, 2000; Greijer et al., 2005). Although HIF-1 usually induces pro survival (CA9, SLC2A1 and VEGF) genes, a role of HIF-1 in regulation of apoptosis has also been described. HIF-1 promotes cell death through an increase in p53 or other proapoptotic proteins like *BCL2*/adenovirus E1B 19 kDa-interacting protein 3 (BNIP3) (Schmid et al., 2004). This dual function of HIF-1α has been shown to maintain a dynamic balance in overall cell growth and survival (Koshiji and Huang, 2004).

### Role of Hypoxia in Cancer Aggressiveness

There are a number of mechanisms through which hypoxia promotes tumor malignancy including; resistance to radio- and chemotherapy (Harris, 2002; Harrison and Blackwell, 2004), increased cell migration and invasion (Fu et al., 2010), reprogramming toward a cancer stem cell (CSC) phenotype and expansion of CSC populations (Heddleston et al., 2009). HIF-1 promotes cell survival in low oxygen conditions by activating the transcription of prosurvival genes (Semenza, 2000) (Figure 2). Moreover, HIF-1 represses *E*-cadherin expression leading to loss of cell–cell adhesion and epithelial-mesenchymal transformation (EMT) through the expression of the transcriptional repressors ZFHX1A, ZFHX1B, and TCF3 (Krishnamachary et al., 2003) and motility through expression of c-MET (Pennacchietti et al., 2003). HIFs regulate the expression and activity of EMT main transcription factors such as Twist, Snail, Slug, Sip1 (Smad interacting protein 1), and zinc finger E-box-binding homeobox 1 (ZEB1) (Jiang et al., 2011). Epigenetic programming can regulate EMT, and EMT may be reversed by dynamic epigenetic modifications (Huangyang and Shang, 2013). Chen and co-workers showed that DNA methylation regulates Snail and Slug genes transcription. The treatment with 5-aza-2′-deoxycytidine, an inhibitor of DNMT, can induce expression of Snail and Slug genes (Chen et al., 2013). Similarly, HIF-1 controls invasion through the expression of matrix metalloproteinase 2, urokinase plasminogen activator receptor and lysyl oxidase (Krishnamachary et al., 2003; Erler et al., 2006). Furthermore, hypoxia has been shown to activate pSTAT signaling and thereby increasing the secretion of immunosuppressive cytokines such as CSF1 and CCL2, which are useful in inhibiting T-cell proliferation and macrophage phagocytosis, which in turn accelerate tumor progression (Wei et al., 2011).

HIF-1 expression has been correlated with tumor grade in gliomas, with the highest expression found in high-grade gliomas like GB (Søndergaard et al., 2002). Pseudopalisading necrosis and vascular proliferation observed in GB tumors is a manifestation of hypoxia and represent tumor cells migrating away from vaso-occlusive, distorted and degenerating blood vessels of the tumor center. As tumor growth is dependent on the formation of new blood vessels, they are often highly vascularized. However, the vasculature is poorly organized and exhibits severe structural and functional abnormalities. Consequently, this leads to regions of the tumor experiencing a reduced supply of oxygen, known as hypoxic regions (Folkman, 1971; Vaupel et al., 2004). Elevated HIF-1α in glioma cells have been shown to cause an increase in the expression of VEGF and CXC chemokine ligand (Jain et al., 2007) both of which promote angiogenesis through different mechanisms, eventually leading to the formation of the malformed vessels, thus leading to a vicious cycle that promotes GB tumor growth and aggressiveness (Würth et al., 2014). Similarly, HIF-1 has also been shown to promote angiogenesis through stromal-derived factor 1 (SDF-1) (Iliopoulos et al., 1996; Krishnamachary et al., 2003).

Twist1 is a basic-helix-loop-helix transcription factor which has been shown to acts as a key regulator of EMT and to promote tumor invasion and metastasis (Jung and Yang, 2015). In this sense, a study that investigated the change of Twist1 expression in human cervical squamous cancer cell line (SiHa) after hypoxia treatment observed that, hypoxia treatment elevated the expression of Twist1 in SiHa cells. Knockdown of Twist1 with siRNA increased the radiosensitivity of SiHa cells under hypoxia condition, accompanied by reduced levels of nuclear Epidermal growth factor receptor (EGFR) and DNA-dependent protein kinase (DNA-PK) (Xiong et al., 2017). Inhibiting the expression of Twist1 could reverse hypoxia-mediated EMT effectively (Sun et al., 2009).

Similarly, hypoxia is a common feature of PCa where hypoxic markers and hypoxia-associated molecules have been associated with a poor prognosis (Stewart et al., 2010). Several reports suggest that hypoxia increases the potential of both resistance and malignancy of PCa (Movsas et al., 2002; Carnell et al., 2006; Milosevic et al., 2007; Vergis et al., 2008). Elsewhere, a study demonstrated an upregulation of the expression a prosurvival gene involved in pH regulation, carbonic anhydrase 9 (CA IX), in three different PCa cell lines namely, PCa-3, Du145 and LNCaP under hypoxia (Fiaschi et al., 2013).

Additionally, TNBC cells can grow, survive, induce metabolic reprogramming and apoptosis, alter cell adhesion and motility to facilitate metastasis and resistance to chemotherapy under hypoxic conditions (Semenza, 2001; Muz et al., 2015). A study done using (MDA-MB-231) TNBC cells showed a translational activation of Integrin beta 3 (ITGB3) under hypoxia and that ITGB3 regulated malignant features, including EMT and cell migration, through the TGF-β pathway (Sesé et al., 2017). Also, hypoxia has been shown to represses the expression of many DNA repair genes including the tumor suppressor gene – BRCA1, which has the important role in preventing the formation of breast cancer (Scanlon and Glazer, 2015). Increased adipose tissue hypoxia can establish a pro-malignancy environment in breast tissues. This was shown by a study that co-cultured human SGBS adipocytes with ER-positive MCF7 breast cancer cells for 24 h and later observed increased expression levels of the EMT-inducing transcription factors FOXC2 and Twist1 (Yao-Borengasser et al., 2015). Additionally, histone demethylase (JMJD2C) has been shown to selectively interact with HIF-1α, but not HIF-2α. Subsequently, JMJD2C decreases trimethylation of histone H3 at lysine 9, and enhances HIF-1 binding to hypoxia response elements, thereby activating transcription of genes encoding proteins involved in metabolic reprogramming for the tumor growth and lung metastasis in breast cancer (Luo et al., 2012).

Although many evidences presented above have been done using cancer cell lines and animal models, clinical evidences also show that HIF-1α accumulation, a factor upregulated during hypoxia, to be associated with poor patient survival in patients with early stage CC (Birner et al., 2000) and lymph node-positive breast cancer (Schindl et al., 2002). Elsewhere an increase in the level of HIF-1α has been shown to correspond with the pathologic stages of breast cancer. Specifically, higher HIF-1α expression was found in poorly differentiated lesions than in the corresponding type of well-differentiated lesions demonstrating that increased levels of HIF-1α are potentially associated with more aggressive tumors (Bos et al., 2001). Significant associations between HIF-2α overexpression and increased patient mortality have been reported for astrocytoma (Khatua et al., 2003).

Based on these studies, it’s clear that hypoxia leads to an upregulation of hypoxia inducible factor culminating in the activation of pro-survival genes or pathways that may help the cancer to acquire a more aggressive phenotype.

### The microRNAs and the Hypoxia Microenvironment

As mentioned earlier, miRNAs regulate the majority of eukaryotic genes and the genes related to hypoxia microenvironment are not an exception. miR-210 is an oncogenic miRNA and a target of HIF-1 and -2 (Gee et al., 2014) whose correlation with hypoxia is a biological phenomenon associated with tumor aggressiveness. It has been shown that during hypoxia, miR-210 targets the mRNA that encodes the mitochondrial electron transport chain component protein succinate dehydrogenase complex subunit D (SDHD). Decreased expression of SDHD results in an increased stabilization of HIF1α and cancer cell survival (Puisségur et al., 2011; Rupaimoole and Slack, 2017). Elsewhere, miR-210 has been shown to downregulate the hypoxia stress response cell death inducer mitochondrion-associated 3 (AIFM3), thereby promoting survival of cancer cells (Wang J. et al., 2014) and ephrin A3, a hypoxia-responsive angiogenesis inhibitor, leading to increased tumor angiogenesis (Fasanaro et al., 2008).

Likewise, HIF-1α subunit, is negatively regulated by the VHL tumor suppressor (Harris, 2002) and miR-155 has been shown to downregulate the expression of VHL tumor suppressor, a protein involved in the cellular response to hypoxia. Additionally, downregulation of VHL has been shown to lead to increased angiogenesis and facilitate cancer cell survival (Kong et al., 2014). Elsewhere, miR-21-5p and miR-23b-3p are also reported to target VHL and decrease the production of the VHL protein, that results to upregulation of vascular endothelial growth factor A (VEGFA) expression (Chen L. et al., 2012; Zhang et al., 2014). In the same sense, miR-7-5p downregulates the expression of *O*-linked *N*-acetylglucosamine transferase (OGT), leading to decreased expression of vascular endothelial growth factor receptor 2 (VEGFR2) (Babae et al., 2014). Notably, overexpression of miR-128 has been shown to downregulate the activity of p70S6K1 and the expression of its downstream signaling molecules such as HIF-1 and VEGF resulting in reduced cell proliferation, tumor growth and angiogenesis (Shi et al., 2012).

In addition, the hypoxic microenvironment also influences the biogenesis of several miRNAs. In this sense, Drosha, a component of miRNA biogenesis, has been reported to be downregulated in response to tumor hypoxia through a process mediated by the direct binding of the hypoxia-responsive transcription factors ETS1 and ELK1 to the promoter of *DROSHA* (Rupaimoole and Slack, 2017). Similarly, Dicer has been shown to be downregulated through direct targeting of the *DICER* 3′ UTR by miRNAs such as miR-103/107 (Martello et al., 2010), *let-7* (Tokumaru et al., 2008) and miR-630 (Rupaimoole et al., 2016) where tumor hypoxia further influenced these effects. Additionally, downregulation of *DICER* expression by epigenetic mechanisms, which are mediated by the hypoxia-induced inhibition of the oxygen-dependent H3K27me3 demethylases KDM6A and KDM6B (van den Beucken et al., 2014), have also been reported.

Table 1 shows a list of miRNAs deregulated under hypoxia microenvironment in the four cancers previously mentioned. Some of the genes involved include VEGFA (angiogenesis) (Yue et al., 2012), WNT1 (proliferation), ERBB3 (proliferation) (Tang et al., 2016), SMAD4 (tumor suppressor) (Phuah et al., 2017), NDRG2 (radioresistance) (Wang W. et al., 2016) and EGFR (invasiveness) (Rupaimoole and Slack, 2017) all leading to tumor aggressiveness. Evidently, there is a connection between miRNAs and hypoxia but further studies are needed.

**Table 1:** microRNAs associated with hypoxia and the stem-like state in four aggressive cancers.

Cancer	Hypoxia	Stem-like state	Reference
Glioblastoma	miR-205-5p, miR-210, miR-21-5p miR-23b- 3p, miR-7-5p	miR-125b, miR-33a, miR-34 *miR-10b*, miR-143, miR-330, miR-582-5p, miR-363, miR-138, miR-153, miR-124, miR-9, miR-128 miR-218, miR-146-a miR-326	Godlewski et al., 2008; Kefas et al., 2009; Li Y. et al., 2009; Li Z. et al., 2009; Guessous et al., 2010, 2013; Shi et al., 2010; Mei et al., 2011; Chan et al., 2012; Chen L. et al., 2012; Yue et al., 2012; Tan et al., 2012; Lee et al., 2013; Qiu et al., 2013; Tu et al., 2013; Zhao et al., 2013a,b; Babae et al., 2014; Floyd et al., 2014; Lai et al., 2014; Wang H. et al., 2014; Yao et al., 2014; Zhang et al., 2014
Cervical cancer	miR-152, miR-210	miR-23b, miR-125b	(Phuah et al., 2017; Tang et al., 2016)(Wang et al., 2013, 2017; Zhou X. et al., 2017)
Prostate cancer	miR-301a/b, miR-517a, miR 204, miR-885, miR-143, miR-335, miR-127, miR-542, miR-433, miR-451, miR-92a and miR-181a miR-521, miR-27a, miR-324, miR-579, miR-502, miR-222, miR-135b, miR-146a and miR-491	miR-141, miR-1301-3p, miR, 150, miR-574	Liu et al., 2015; Liu C. et al., 2017; Chen et al., 2016; Wang W. et al., 2016; Panigrahi et al., 2018; Song et al., 2018
Breast cancer	miR-210, miR-155	miR-590-5p, miR-181a-5p, miR-30e-5p, miR-34	Choi et al., 2011; Kong et al., 2014; Rupaimoole et al., 2016; Liu K. et al., 2017; Zhou L. et al., 2017

*Several microRNAs have been associated with the hypoxia microenvironment and the cancer stem-like state. Their roles are not fully understood but they all play roles that favor the tumor aggressiveness. miR, microRNAs*.

## The Cancer Stem-Like State

Cancer stem-like cells, also known as tumor-initiating cells or tumor-propagating cells, are a subpopulation of tumor cells that selectively possess tumor-initiation and self-renewal capacity and the ability to give rise to bulk populations of non-tumorigenic cancer cell progeny through differentiation. They propagate tumors phenotypically similar to the parental tumor (Chen J. et al., 2012).

### The Stem-Like State and Cancer Aggressiveness

Tumors comprise a heterogeneous cell population, with 0.1–0.8% of these tumor cells being cancer stem cells (Konrad et al., 2017). CSCs share similar features with the normal stem cells such as expression of the stem cell markers, capacity for self-renewal and long term proliferation, formation of tumor sphere among others. In contrast, solid cancer stem cells differ from normal stem cells in frequency, proliferation, aberrant expression of differentiation markers, chromosomal abnormalities and tumor formation (Reya et al., 2001; Nimmakayala et al., 2018). CSCs have been implicated in the initiation, progression, maintenance and recurrence of tumors in a variety of cancers (Reya et al., 2001; Singh et al., 2004; Vander Griend et al., 2008; Yi et al., 2008; Tu and Lin, 2012; Xie et al., 2016). The potent tumor initiation of cancer stem cells together with their radioresistance (Debeb et al., 2009), and chemoresistance (Li et al., 2008) suggests that these cells contribute to tumor aggressiveness (Figure 2).

Epigenetic mechanisms have been shown to play a role in the pathogenesis of CSCs (Shukla and Meeran, 2014; Li and Li, 2015). Specifically, DNA methylation plays an important role in the loss of pluripotency and developmental plasticity in cells. In addition, CSCs can be generated from epigenetic reprogramming where specific genes of stem cells recover their expression, while specific genes for differentiation are downregulated (Shukla and Meeran, 2014). The Wnt/β-catenin pathway plays important functions in self-renewal and differentiation of CSCs (Hoffmeyer et al., 2012). DNA methylation has been linked to aberrant Wnt/β-catenin pathway activation through the enhanced promoter methylation and subsequent silencing of various Wnt inhibitors such as *WIF-1, AXIN2*, *SFRP-1* and *DKK1* in breast cancers (Klarmann et al., 2008). Activation of EMT can confer cells with CSC and tumor-initiating properties (Morel et al., 2008) and loss of membrane protein *E*-cadherin is a hallmark of EMT. DNA methylation of E-cadherin promoter has been shown to help recruit HDACs to the site, leading to histone deacetylation and its transcriptional silencing (Wang and Shang, 2013). Transcriptionally active chromatin marked by H3K9Ac has been shown to be reinforced by H3K4 methylation (H3K4me3) and H3K9 hyperacetylation to induce the expression of OCT4 and Nanog to maintain self-renewal (Chen and Daley, 2008). Likewise, CD44, CD133 and Musashi-1 promoters presented a hypomethylated status which was associated with high expression of CSC markers in TNBC (Kagara et al., 2012). The studies discussed above are selected examples illustrating how epigenetic mechanisms control the transcription control of CSC in various malignancies. Aberrant epigenetic mechanisms may transform normal stem cells to cancer stem cells with the loss of differentiation capacity and the acquisition of stem-like phenotypes that help to maintain malignant growth.

In addition, different clinical studies have sought to link the presence of cancer stem cell-like state to cancer development, progression and patient prognosis. A study by Tao et al. comparing the levels of expression of cancer stem cell-state markers ALDH1, CD117 and CD133 in 52 ovarian cancer patients showed that increased levels of these markers were significantly associated with higher tumor grade (Tao et al., 2018). Another study retrospectively analyzed serum samples from 140 patients for the expression of CD44 and ALDH1, the major markers of stem cell state in breast cancer. They found out that patients with a higher expression of CD44 had a significantly shorter overall survival and progression free survival compared to those with lower expression (Kong et al., 2018). In CC, a study analyzing the samples of 139 patients found a correlation between a low expression of P16INK4A, a blocker of stem cell self renewal ability, and high expression of ALDH1 with poor outcomes in patients treated with radiotherapy with/without chemotherapy (Fu et al., 2018). This was in agreement with another study by Lin et al. that showed the over expression of P16INK4A was correlated with better prognosis in CC by down regulating reprogramming of cells into the stem cell state (Lin et al., 2014). Other studies have shown variable associations of cancer stem cell state markers with progression of different cancers. For example, the expression of CD133 and CD44 in GB patients was shown to be negatively correlated with the survival time (Metellus et al., 2010; Pietras et al., 2014). Similarly, a study found OCT4 to be over-expressed in CC tissues as compared to the adjacent normal tissues. Moreover, high OCT4 expression was positively associated with radiotherapy resistance and an independent risk factor for CC (Yang et al., 2014). Likewise, an immunohistochemistry analysis study, aiming to compare the expression of Nanog in patients with CC and cervical dysplasia in 235 patients with various degrees of cervical epithelial lesions, found significantly higher expression of Nanog in CC than in cervical dysplasia and higher in cervical dysplasia than in normal cervical epithelia (Ye et al., 2008). Therefore, these studies provide evidences for the clinical relevance of CSCs.

### The Stem-Like State and Hypoxia

Low oxygen tension is associated with maintenance of an undifferentiated cell state and it has been shown to promote the self-renewal of embryonic stem (ES) cells and prevent the differentiation of stem cells *in vitro* (Parmar et al., 2007; Clarke and van der Kooy, 2009; Silván et al., 2009). Several studies have shown that restricted oxygen conditions expand the fraction of cells positive for cancer stem cell markers (Tavaluc et al., 2007; Das et al., 2008; McCord et al., 2009). In this sense, a study done by Heddleston and group demonstrated that extended exposure to hypoxia can result in a phenotypic shift in the non-stem population to mirror that of the stem-like subset and promote cell growth and self-renewal. They also found out that non-stem cells cultured under hypoxia were able to form oncospheres at twice the rate of control cells in normoxia. Moreover, following long term-culture (10–14 days) of non- stem cells in hypoxia, the OCT4, Nanog and c-Myc displayed significant and consistent increase in T4121 non-stem glioma cells as compared to normoxia (Heddleston et al., 2009).

Similarly, a study observed that CSCs under hypoxic conditions displayed an increased VEGF expression relative to the non-stem cells. In addition, CSCs specifically regulated several targets (HIF-2α and transcriptional targets of HIF-2α: OCT4, Glut-1 and Serpin B9) under hypoxia to a greater degree than non-stem cells (Li Z. et al., 2009).

In the same sense, a study done by Li and group, using primary glioma cells, showed that hypoxia maintained the cells in undifferentiated state, slowed their growth by entering into quiescent phase and increased their colony forming efficiency and migration. The cells had elevated expression of stem cells markers and decreased expression of markers associated with differentiation (Li et al., 2013). Similarly, hypoxia has also been shown to increase the expression of stem-like cell markers and to promote clonogenicity in neurospheres from GB (Bar et al., 2010).

Accordingly, expression of HIF-1α, Nanog and OCT4 has been observed in primary PCa tumors, of which, expression of Nanog and OCT3/4 were highly enriched in HIF-1α positive tumor regions. A different study also suggested SOX2 as a hypoxia-responsive gene that contributes to PCa cell invasion and sphere formation mediated by low oxygen tensions (Bae et al., 2016). Elsewhere, a study done using MDA-MB-231, a representative TNBC cell line, showed an increased proportion of stem cells population and a significantly increased cell colony-formation rate in MDA-MB-231 cells after they were treated with hypoxia (Xie et al., 2016). Similarly, Samanta and colleagues demonstrated that chemotherapy-induced HIF activity enriched the TNBC stem cell population through interleukin-6 and interleukin-8 signaling and increased expression of multidrug resistance 1. Moreover, they showed that increased expression of HIF-1α or HIF target genes in breast cancer biopsies was associated with decreased overall survival (Samanta et al., 2014).

There is clear evidence showing hypoxia modulates several types of cancers, however, this topic deserves more attention especially in cervical and breast cancers.

### The microRNAs and Cancer Stem-Like State

There is an established relationship between miRNAs and the cancer stem-like state. In this sense, the EMT-activator ZEB1 is a crucial promoter of metastasis that has been shown to repress the expression of stemness-inhibiting miR-203. Also, the candidate targets of the miR-200 family have been shown to be stem cell factors, such as SOX2 and KLF4. Moreover, the miR-200c, miR-203, and miR-183 have been shown to suppress the expression of stem-like cell factors in cancer cells and mouse embryonic stem (ES) cells, by targeting the polycomb repressor Bmi1 (Wellner et al., 2009).

Glioblastoma stem-like cells (GSCs) share some characteristics with normal neural stem-like cells, such as expression of neural stem cell markers, capacity for self-renewal and long-term proliferation, formation of neurospheres and the ability for multi-lineage differentiation into nervous system lineages (neurons, astrocytes and oligodendrocytes) (Vescovi et al., 2006). In GSCs obtained from human U251, miR-125b was the first miRNA found to be downregulated and shown to play a role in GSC maintenance (Shi et al., 2010; Zhao et al., 2014). Since then, several others miRNAs have been related with stem-like state feature in GB. For example, overexpression of miR-33a in non- GSC glioblastoma cells promoted the display of features associated with GSCs, suggesting that this miRNA contributed directly to the GSC phenotype (Wang H. et al., 2014). Elsewhere, suppression of reprogramming by miR-34a was due, at least in part, to repression of pluripotency genes, including Nanog, SOX2 and Mycn (N-Myc). A different study also showed that miR-34a directly inhibits the expression of Notch-1, Notch-2 and c-Met in GSCs (Li Y. et al., 2009; Guessous et al., 2013). Additionally, miR-10b is upregulated in human GB and GSC lines and its inhibition strongly reduces the proliferation, invasion and migration of GSCs. Moreover, miR-10b inhibition has been shown to significantly decrease the growth of GSC-derived orthotopic GB xenografts (Guessous et al., 2013).

Elsewhere, over-expression of miR-143 was shown to inhibit glycolysis, promote differentiation, and decrease the tumorigenic capacity of GSCs *in vivo*, highlighting its important role as a tumor suppressor (Zhao et al., 2013b). OncomiR-138 has been identified as part of a molecular signature and prognostic biomarker of GSCs (Chan et al., 2012). Similarly, miR-330, miR-582-5p, and miR-363 have been shown to promote GSC migration and invasion by reducing apoptosis (Floyd et al., 2014; Yao et al., 2014). Also, transient transfection of miR-153 into GSCs was shown to impair their self-renewal ability and induce their differentiation. Moreover, miR-153 could also repress tumor stem cell growth and induce apoptosis (Zhao et al., 2013a). The transcription factor, SOX2, is essential for maintaining stem cell self-renewal and pluripotency and is a direct target of miR-124 therefore, miR-124 has been shown to decrease the migration of GB cells and inhibit self-renewal of GSCs by targeting SOX2 (Lee et al., 2013). Several other miRNAs that regulate proliferation, self-renewal and migration of GSCs have been described, including miR-9 (Tan et al., 2012), miR-128 (Godlewski et al., 2008), miR-218 (Tu et al., 2013), miR-146-a (Mei et al., 2011), and miR-326 (Kefas et al., 2009) miR-21 is regarded as an onco-miR in GB and its over-expression has been associated to poor prognosis. Surprisingly, the pro-differentiation role of miR-21 over-expression in GSCs has been shown (Zhi et al., 2010). In this regard, findings from a study demonstrated that the involvement of miR-21 in the positive regulation of differentiation of GSCs suggested that miR-21 inhibition could increase the stemness, a probable source of tumor relapses after GB treatment (Aldaz et al., 2013) (Table 1).

ALDH1A1, intracellular enzyme with detoxifying role, has been identified as cancer stem cells marker for CC (Douville et al., 2009). In this regard, a study showed that miR-23b was down-regulated in Hela and CaSki cervical cancer stem cells (CCSCs) derived from tumor spheres and that miR-23b directly binds to the 3′UTR of ALDH1A1 to suppress its translation. Importantly, targeted expression of miR-23b was shown to disrupt the stemness of CCSCs while miR-23b inhibition increased the population of cervical tumor spheres (Wang et al., 2017). The miR-145 has been shown to induce the differentiation of CSC by down-regulating the stem cell transcription factors that maintain CSC pluripotency. In this sense, high expression of miR-145 indicates a good prognosis in CC patients and a therapeutic target (Zhou X. et al., 2017). The transcription factor OCT4 has also been shown to directly induce the expression of miR-125b, by inhibiting its direct target, BAK1, leading to suppression of CC cell apoptosis (Wang et al., 2013).

Accordingly, miR-141 also suppresses prostate cancer stem cells (PCaSCs) and its metastasis by targeting a cohort of pro-metastasis genes (Liu C. et al., 2017). Elsewhere, a study found out that miR-1301-3p promoted the expansion of PCaSCs by targeting the inhibitors of Wnt pathway, GSK3β and SFRP1, and consequently activating the pathway (Song et al., 2018). Another pathway explored for PCaSCs maintenance is the suppression of p27, caused for instance, by increased miR-150 expression, that might participate in the development and progression of PCaSCs (Liu et al., 2015). It has also been shown that downregulation of miR-34c in cancer stem cells causes recurrence of PCa (Chen et al., 2016).

Regarding breast cancer, Zhou and team showed that miR-590-5p inhibited BC cells stemness through targeting SOX2 and they suggested that miR-590-5p might be a useful strategy for BC treatment (Zhou L. et al., 2017). Also, shRNA-mediated knockdown of SOX2 has been shown to inhibit BC cell expansion and migration followed by a significant reduction in the levels of miR-181a-5p and miR-30e-5p. Moreover, overexpression of these two miRNAs resulted in a reduced protein level of the tumor suppressor candidate 3 (TUSC3) in BC cells. Consequently, mutations of the potential binding sites in the 3′-UTR of TUSC3 abrogated the inhibitory effects of the miRNAs (Liu K. et al., 2017). Elsewhere, a study done by Choi et al. (2011), identified miR-34 as a p53 target that plays an essential role in restraining somatic reprogramming and genetic ablation of miR-34a promoted iPSC generation without compromising self-renewal or differentiation (Choi et al., 2011) (Table 1).

Therefore, miRNA based therapy targeting CSCs has the potential to allow more effective treatment strategies in the future.

### miRNAs, Hypoxia and Stem-Like State and Future Therapy Prospects

The main aim of miRNA therapeutic modulation is to design molecules that can inhibit or mimic mature miRNAs effectively in order to reverse the loss or gain of miRNA function. Based on this concept, targeting miRNAs overexpressed in cancer or re-expressing those downregulated in cancer represents an optimal approach and both the stem-like state and the hypoxia microenvironment might be a target as well.

The inability to reduce the HIF-1 levels during prolonged hypoxia leads to cell death, making this a potential therapeutic target in cancer. To escape this death during continuous oxygen depletion, cells pass the signal from HIF-1 to HIF-2 and eventually to HIF-3 thus allowing them the ability to promote angiogenesis and long-term survival through a process known as the HIF switch (Serocki et al., 2018). Importantly, miRNAs can regulate the HIF switch during hypoxia making them important therapeutic targets (Serocki et al., 2018). In this sense, a study showed an upregulation of a miR-18a after 24 h in hypoxia a direct target of HIF1A mRNA (Jiang, 2015) suggesting its ability to induce to the HIF switch. Elsewhere, miRNA-mediated beta subunits suppression has been shown to limit HIF-1 activity and angiogenesis providing a novel, alpha subunit-independent mechanism for HIF signaling regulation (Yamakuchi et al., 2010; Deng et al., 2016). In human CC (HeLa) cells, miR-147 has been shown to reduce the levels of HIF-3 isoform, a dominant-negative regulator of HIF-1, in turn stabilizing HIF-1 accumulation (Wang F. et al., 2016).

Moreover, HIF-1α is activated in normal neural progenitors and is present in both CSC and non stem-like cells at more severely hypoxic conditions (≤1%) limiting its value in terms of therapeutic targeting (Li Z. et al., 2009). However, HIF-2α offers hope as a therapeutic target in GB in that it is practically absent in non-glioma stem-like cells and specifically elevated in GSCs, even under modest hypoxic conditions (Carmeliet and Jain, 2000; Cárdenas-Navia et al., 2008; Li Z. et al., 2009). The regulation of HIF2α is not a general stem cell phenomenon as normal neural progenitors express essentially no HIF2α mRNA or protein.

Besides this, many HIF inhibitor molecules have been identified (Xia et al., 2012; Serocki et al., 2018) but none of the presently available inhibitors appears to disrupt the HIF-1 pathway exclusively consequently resulting to regulation of a wide range cellular metabolic activities (Rodriguez et al., 2016). However, recent efforts has been made to develop selective HIF-2 inhibitors that are now in clinical studies (Wallace et al., 2016).

The development of therapies against CSCs is challenging because both bulk cancer cells and CSCs must be eliminated and so far it has proven difficult. Similarly, CSC-targeted therapy may damage normal stem/progenitor cells and block the regeneration of normal tissues, causing tissue or organ dysfunction (Huang and Rofstad, 2017). However, cell based delivery of miRNAs or miRNA inhibitors offer a promising approach as it can abolish CSCs by modulating their function and/or expression of important proteins to at least that of non-CSCs. Therefore as discussed under ‘stem-like cells and miRNAs’ section, the same miRNAs implicated in the regulation of the CSC properties can be targeted using miRNA modulation studies to reverse the cancer stem cell fate.

As epigenetic mechanisms have important functions in modulating miRNA expression, cancer progression and stem cell properties in cancer cells, targeting components of these epigenetic pathways would help in reversing this fate. A summary of these epigenetic drugs and their clinical status can be found in Toh et al. (2017). Similarly clinical trials using miRNAs as therapeutic targets have been summarized by Chakraborty et al. (2017) and Rupaimoole and Slack (2017). Specifically, MRX34 (Mirna Therapeutics) have completed a multicentre phase I trial by using miR-34 mimic to target multiple solid tumors (ClinicalTrials.gov identifier NCT01829971).

Although many important questions regarding miRNAs biological function, target specificity, stability, safety, efficacy and effective delivery remain to be addressed, the possibility that miRNAs or their specific target protectors can be used in future therapeutic approaches, to reverse the loss and gain of functions, to regulate the hypoxic HIF switch, to regulate hypoxic microenvironment and stem cell reprogramming, emphasizes the importance of the interconnect relationship between the miRNAs, hypoxia and stem-like state to the cancer aggressiveness.

## Conclusion

Tumor therapy resistance, invasiveness and poor patient survival are features shared by several aggressive cancers. The miRNAs, hypoxia, hypoxia inducible factors and the stem-like state all play key roles in contributing to the cancer aggressiveness. Importantly, miRNAs are often found dysregulated in various tumors such as glioblastoma, breast, prostate, and cervical cancers where they regulate several processes and pathways contributing to tumorigenesis angiogenesis, growth, infiltration and resistance. Several miRNAs have been shown to play critical roles in the interactions between the tumor and the tumor microenvironment and specifically, they regulate several key genes and signaling pathways that lead to adaptation of the cancer cell in hypoxic conditions. In like manner, hypoxia regulates a set of miRNAs commonly referred as HRMs and even influences the biogenesis of miRNAs. This interaction between miRNAs and hypoxia or HIFs can account for many vital events relevant to tumorigenesis such as angiogenesis, metabolism, apoptosis, cell cycle regulation, proliferation, metastasis, stemness and resistance to anticancer therapy. Both hypoxia and the miRNAs have similarly been implicated in the maintenance of CSCs that drive the tumor propagation ability and contributes to the tumor resistance. Therefore, based on the evidence covered in this review, it is possible to conclude that miRNAs, hypoxia and the stem-like state play a connected role, like a feedback loop or a complex signaling network in promoting tumor aggressiveness and future miRNA targeted therapies should factor in these concepts.

## Author Contributions

All authors conceptualized, wrote, edited and critically evaluated the manuscript, and also approved the final version of the manuscript. LM prepared the final version of the manuscript. VF and VM-N supervised the work.

## Conflict of Interest Statement

The authors declare that the research was conducted in the absence of any commercial or financial relationships that could be construed as a potential conflict of interest.

## Abbreviations

5-mC: 5-methylcytosine
ACC: cervical adenocarcinoma
ADT: androgen deprivation therapy
AGO: argonaute
AMOs: antisense miRNA oligonucleotides
AR: androgen receptor
*ATM*: ataxia-telangiectasia
BP: base pairs
BRCA1: Breast cancer 1
CA125: Carbohydrate antigen 125
CC: cervical cancer
*CHEK2*: checkpoint kinase 2
CIN: cervical intraepithelial neoplasia
CSC: Cancer stem-like cells
DGCR8: DiGeorge syndrome critical region 8
DHT: dihydrotestosterone
DNA: deoxyribonucleic acid
DNMTs: DNA methyltransferases
dsRBPs: dsRNA-binding proteins
EGFR: epithelial growth factor receptor
ELF: eukaryotic initiation factors
EMT: epithelial to mesenchymal transition
ER: estrogen receptor
Exp5: exportin 5
FFPE: Formalin fixed paraffin –embedded
GB: glioblastoma
*GST*: Glutathione S-transferase
GW182: glycine tryptophan protein of 182 kDa
H3K27me3: trimethylated histone H3 lysine 27
HATs: histone acetyltransferases
HDACs: histone deacetylases
HER2: human epidermal growth factor receptor type 2
HIF: hypoxia indicible factor
HNPRK: heterogeneous nuclear ribonucleoprotein K
HRE: HIF responsive element
HRMs: hypoxia- regulated miRNAs
*IDH*: isocitrate dehydrognase
*IFNG*: interferon, gamma
IGF: insulin growth factor
IPSC: induced pluripotent stem cells
KGF: Keratinocyte growth factor
lnRNA: long non-coding RNA
MEF: mouse embryonic fibroblast
MGMT: O(6)-methylguanine-DNA methyltransferase
miRISC: miRNA-induced silencing complex
miRNAs: MicroRNAs
NTS: Nucleotides
OCT4: Octamer-binding transcription factor 4
ODDD: oxygen-dependent degradation domain
PABP: poly A-binding protein
PAZ: Piwi, Argonaute and Zwille
PCa: prostate cancer
PHD: prolyl hydroxylases
piRNA: Piwi RNA
PR: progesterone receptors
pre-miRNA: precursor miRNA
pri-miRNA: primary miRNA
PSA: prostate specific antigen
PSCA: prostate stem cell antigen
PTEN: phosphatase and tensin homolog
SCC: squamous cell carcinoma
SDF-1: Stromal derived factor 1
siRNA: Small interfering RNA
SOX2: SRY-like HMG-box gene
TARBP: Transactivation responsive RNA binding protein
TERT: telomerase reverse transcriptase
*TMC6/8*: transmembrane channel-like 6 and 8
TNBC: tripple negative breast cancer
TP53: tumor Protein 53
UTR: untranslated region
VEGF: vascular endothelial growth factor
VHL: von Hippel –Lindau
VMP1: Vacuole membrane protein 1
ZEBI: Zinc finger E-box binding homeobox 1
